# How Air Quality Affect Health Industry Stock Returns: New Evidence From the Quantile-on-Quantile Regression

**DOI:** 10.3389/fpubh.2021.789510

**Published:** 2021-12-23

**Authors:** Lu Liu, Kai-Hua Wang, Yidong Xiao

**Affiliations:** ^1^School of Management, Ocean University of China, Qingdao, China; ^2^School of Economics, Qingdao University, Qingdao, China; ^3^Graduate School of Economics, The University of Tokyo, Tokyo, Japan

**Keywords:** air quality, stock return, health industry, quantile-on-quantile method, heterogeneity

## Abstract

This paper discusses the asymmetric effect of air quality (AQ) on stock returns (SR) in China's health industry through the quantile-on-quantile (QQ) regression method. Compared to prior literature, our study provides the following contributions. Government intervention, especially industrial policy, is considered a fresh and essential component of analyzing frameworks in addition to investors' physiology and psychology. Next, because of the heterogeneous responses from different industries to AQ, industrial heterogeneity is thus considered in this paper. In addition, the QQ method examines the effect of specific quantiles between variables and does not consider structural break and temporal lag effects. We obtain the following empirical results. First, the coefficients between AQ and SR in the health service and health technology industries change from positive to negative as AQ deteriorates. Second, AQ always positively influences the health business industry, but the values of the coefficients are larger in good air. In addition, different from other industries, the coefficients in the health equipment industry are negative, but the values of the coefficients change with AQ. The conclusions provide important references for investors and other market participants to avoid biased decisions due to poor AQ and pay attention to government industrial policies.

## Introduction

Human health, which is one of the essential dimensions of and signs in sustainable development, is the comprehensive embodiment of social, economic, and physical conditions (1, 2). However, human health is now confronting great challenges from the air quality problem, and the vast majority of the literature has confirmed this adverse effect (3–6). In 2015, more than 9 million premature deaths worldwide were due to air pollution, which is much higher than the number of deaths from wars and other violence (7). In particular, air pollution influences human health through acute health effects, chronic health effects, and intervention effects (8). In addition to health problems, air pollution also results in total welfare losses and has a negative influence on economic activities (8, 9). The health care costs triggered by air pollution are expected to reach 176 billion U.S. dollars in 2060, and 3.7 billion working days are expected to be lost. In addition to the traditional focus on human health and economic performance, more attention has been given to financial markets, especially stock markets. Air pollution changes investors' moods and their trading behaviors and finally has a negative influence on stock returns (SR) (10–12). Prior studies have mainly focused on air pollution and emphasized that SR is impacted by poor air quality (AQ). However, investors and other participants cannot be affected by only polluted air and should further consider the effects of good AQ. The good weather, such as sunshine, has been proved to be positively correlated with stock returns in London, Singapore, New York, and other major stock markets (13, 14). As for good air quality, it would make people feel optimistic relax for future economic performance (15), and tend to risk-taking behaviors, which changes their trading activities in stock market (16–19). Our paper depends on AQ data and the quantile-on-quantile method to capture heterogeneous responses from stock returns to healthy and unhealthy air quality at the same time, which could provide more valuable referential policy implications for market participants.

There are multiple explanations for the relationship between AQ and SR in China's health care industry. First, China is a rapidly developing country and suffers serious environmental problems (20–23). In 2020, China emitted 9.89 billion tons of carbon dioxide (CO_2_), 3.7 million tons of sulfur dioxide (SO_2_), and 11.57 million tons of nitrogen oxide (NOx). Large amounts of CO_2_, SO_2_, NO_x_, and other pollutants influence the environment and further lead to haze and fog, acid rain and other problems. In 2019, China's average PM_2.5_ concentration was 39.1 μg/m^3^, which was not only higher than those of the U.S. (9.01 μg/m^3^) and the U.K. (16.6 μg/m^3^) but also exceeded those of Brazil (15.77 μg/m^3^) and Malaysia (19.36 μg/m^3^). Second, air quality, especially air pollution, is an urgent public health problem. Exposure to air pollutants such as PM_2.5_ and SO_2_ would increase the short-term and long-term risks for diseases (24). For example, exposure to PM2.5, PM10, NO_2_, and SO_2_ results in emergency hospital visits of 3.34, 3.96, 5.90, and 5.38%, respectively (25). Heyes and Zhu (26) also show that one standard deviation increases in AQ index and PM_2.5_ were associated with ~11.6 and 12.8% increases in sleeplessness, respectively. In addition, in cities of Shanghai (27), Dongguan (28), Shenzhen (29), Hefei (30) and others, the health impacts of air pollution are also found significantly. Although there are apparently many benefits resulting from decreased air pollution, we still cannot address the problem of air pollution effectively. Third, the serious consequences of air pollution are not limited to human health, but also spread to economic fields. Some studies have proved that air pollution changes investors' mood and make them do biased decisions, and finally affects the stock market (12, 19, 31–34). In addition, the existing literature (10, 13) almost focus on the effect of air pollution on stock return in developed states and regions. Given the serious problem of air pollution more happens in undeveloped countries and regions, significant irrational character in their stock markets, and directly relationship with air quality, we are trying to examine the asymmetric effect of AQ on SR in China's health industry and offer valuable references for investors.

This paper discusses the link between AQ and SR across different health care industries, and we obtain the following results. First, the coefficients between AQ and SR in the health service and health technology industries change from positive to negative as AQ deteriorates. Second, AQ always positively influences the health business industry, but the values of the coefficients in the [0, 0.5] quantile are larger than those in other quantiles. In addition, different from former industries, the coefficients in the health equipment industry are negative, but the values of the coefficients change as AQ changes.

Compared to previous studies, this paper offers some meaningful contributions in discussing the asymmetric effect of AQ on SR in China's stock markets. Government intervention, especially industrial policy, is considered a new and indispensable aspect in explaining the effect of AQ on SR. Prior studies commonly choose investor mood as a major transmission mechanism for interpreting the influences of AQ (18, 19, 35–37). However, government intervention has been proved to produce significant influence on stock market (38–40), especially in China (41–43). For example, China is implementing supply-side reform, which aims to reduce excess capacity and optimize the national industrial structure. Therefore, government intervention should be considered since it can better explain the effect of AQ on SR in China's stock markets. Next, the heterogeneous responses of different health care industries to air quality are sufficiently considered in this paper. Previous studies commonly employ stock market indices, such as the S&P Composite Index, to discuss the relationship between AQ and SR (10, 31, 36, 44, 45). Thus, in order to obtain more accurate results, four subdivided industrial indices, including health services, health equipment, health businesses, and health technology, are chosen as our sample. This can better recognize the heterogeneous responses from different health industries to different AQ levels. Finally, we apply the quantile-on-quantile (QQ) method to explore how air quality influences stock returns across different quantiles. This method combines non-parametric estimation and traditional quantile regression, which can recognize the effects between variables in specific quantiles without considering structural break and temporal-lag effects (46, 47). Thus, our study focuses on the asymmetric characteristic of the relationship between AQ and SR using the QQ method and obtains more comprehensive results.

The remainder of our paper is constructed as follows. Section Literature Review is the literature review. Section Transmission Mechanism presents the transmission mechanism. Section Quantile-on-Quantile Regression introduces the quantile-on-quantile method. Sections Data and Empirical Results presents the data and empirical results, respectively. Section Conclusion and Policy Implications summarizes the conclusion and policy implications.

## Literature Review

### Air Quality and People's Health

The relationship between air pollution and health problems has been found in numerous studies (48). Torres et al. (49) indicate that air pollution is related to problems in the cardiovascular and respiratory systems and other adverse side effects. Tainio et al. (50) suggest that air pollution would reduce physical activity levels or hinder people from joining physical activity during heavy pollution episodes. Dominski et al. (51) show that long-term and direct exposure to air pollution, such as PM_2.5_ and PM_10_, would raise the morbidity and mortality of the population. Giaccherini et al. (52) discover that air pollution leads to an increase in hospitalizations; and the effect is stronger among older adults, less educated individuals and migrants. Ma et al. (53) show that reducing PM_10_ and PM_2.5_ is considered the major way to relieve human health damage, and increased CO_2_ levels are responsible for environmental damage. However, Dimitriou and Christidou (54) and Ngo et al. (55) demonstrate that there is no obvious relationship between air pollution and human health for residents.

For China, some studies have also started to focus on the link between air quality and health (56–58). Chen et al. (59) show that the air pollution from traffic has an obvious negative influence on health status among Shanghai residents. Voorhees et al. (60) indicate that the estimated avoided deaths due to air pollution has increased from 13~55 cases per year to 300~800 cases per year in Shanghai. Chen et al. (4) offer evidence for the adverse impacts of air pollution and its spatial spillover effect on public health. Yang and Liu (61) discover that life satisfaction is positively related to health, and this link is influenced by the effect of air pollution. Huang et al. (62) show that air pollution is correlated with deaths, and the effects of both daily and durational air pollution can explain the increased disease burden. Chen and Chen (63) demonstrate that individuals with high incomes, high education and health insurance are more vulnerable to air pollution. Liao et al. (64) demonstrate that sleep loss due to air pollution has a negative influence on human fitness and further increases personal health costs. Liu and Hu (65) show that air quality has a significant impact on residents' life satisfaction, and both the main pollutants and the overall air quality have a significant negative impact. Wang et al. (43) discover that the COVID-19 induced lockdown improves air quality, and the expected averted premature deaths due to air pollution declines are around 26,385–38,977 during the sample period. Zhou et al. (66) indicate that the health risk of preterm birth is positively related to exposure to air pollutants, especially PM_10_, PM_2.5_, and SO_2_.

### Air Quality and Stock Return

With the increasing prominence of air pollution and awareness of environmental protection, scholars have gradually paid more attention to the link between air pollution and stock markets. Levy and Yagil (10) show that air pollution is negatively related to stock returns, but the link weakens as the distance from the polluted area increases. Lepori (31) argues that air pollution deteriorates individuals' moods and intensifies their risk aversion, which reduces demand and lowers stock returns. Kim and Yoo (67) discover that the effect of cumulative exposure to air pollutants on stock returns and volatility is greater than that of daily exposure. Nguyen and Pham (34) discover that stock market anomalies become more significant when severe air pollution occurs because air pollution accelerates behavioral biases. However, Andrikopoulos et al. (68) hold a different opinion that mood changes caused by the weather do not significantly affect stock returns.

Because of increasingly serious air pollution issues, a number of studies have started to pay more attention to the effects of air quality on China's stock markets. Zhang et al. (11) demonstrate that air pollution has a negative influence on stock returns and a positive effect on stock volatility. An et al. (36) find that air quality and investor mood are able to influence stock markets separately or jointly. Wu et al. (69) present that air pollution affects investors' sentiment, which makes investors feel pessimistic regarding future stock performance and results in a decrease in stock returns. Wu et al. (70) demonstrate that poor air quality brings low returns, turnover, and volatility, which mainly result from home bias. Ko et al. (71) indicate that when the economy is depressed, investors' emotions are more easily influenced by air pollution. Wu et al. (12) show that air pollution leads to a pessimistic mood among stock traders, which influences the prices of their shareholdings. Teng and He (72) indicate that environmental awareness would produce different influences on investor trading behaviors and corresponding stock prices under different air pollution levels. Liu et al. (73) find that air pollution is negatively related to the stock prices of polluting companies and positively related to the stock prices of new energy companies. Xu et al. (74) show that public awareness plays an essential intermediate role in connecting air pollution and stock returns. Ding et al. (75) show that listed companies present lower stock returns where they are located in cities with higher levels of air pollution. Although many studies argue that there is a negative relationship, some other studies hold different opinions. He and Liu (18) show that the effect of air pollution on China's stock market is non-significant over a long-term period. In addition, new research results are proposed to supplement the existing literature. Li and Peng (17) show that there is not only a contemporaneous negative link between air pollution and stock returns but also a 2-day lagged positive link. Giudici et al. (76) indicate that events and incidents related to air pollution produce obvious positive or negative stock returns, which depend on the industry.

Although many studies discuss the link between air quality and stock returns, research gaps remain. Few studies focus on China's health industry and its subdivided sectors, including health service, health equipment, health business, and health technology. Traditional econometric methods, including vector autoregression and Granger causality, do not perform well in capturing the non-linear characteristics of the link. Therefore, the quantile-on-quantile method is employed to recognize the varying degree of the influences. In addition, our study takes the effects of different quantiles of air quality into consideration, which could yield novel conclusions.

## Transmission Mechanism

**Figure 1:**
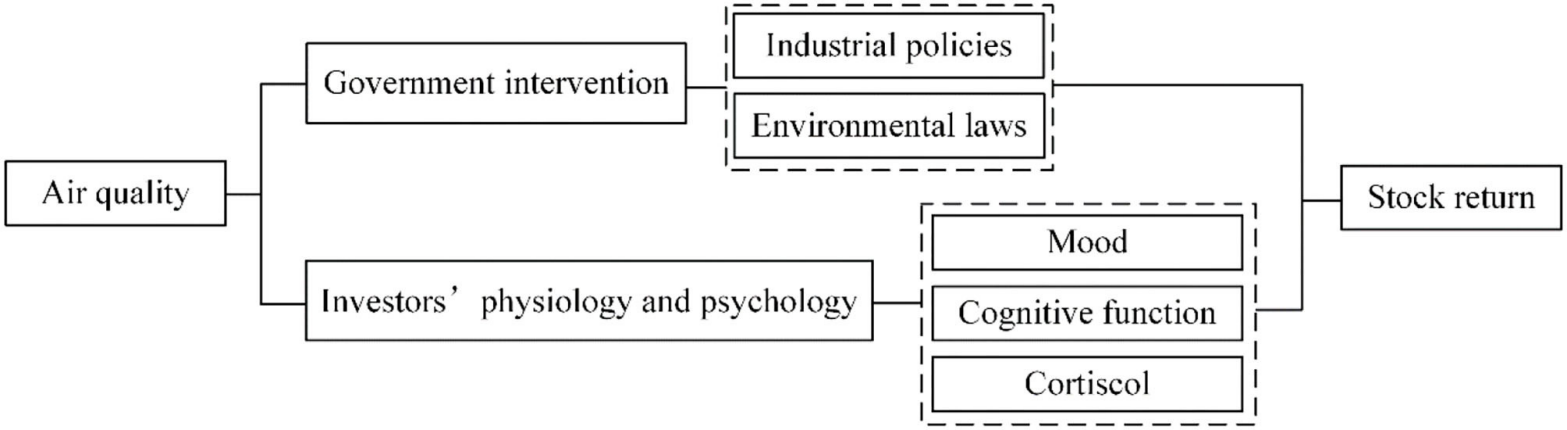
The transmission mechanism.

Many studies indicate that air pollution can influence the stock market through multiple channels, which are shown in Figure 1.

The first channel is that air pollution can lead to government intervention, especially industrial policies and environmental protection laws (77). Given the serious human health damage and welfare loss caused by air pollution, governments confront great social pressure to handle air pollution (25, 42, 78–80). China's government has published a series of policies to support the development of the health care industry. For example, China released a document called the *Development Plan of the Health Equipment Industry (2021–2025)*, which clearly establishes the goals that high-end health equipment will be safe and reliable, product performance and quality will reach international levels, and the health equipment industry system will be basically perfect by 2025. Positive and supporting policies will boost market confidence, affect people's expectations for companies' performance, and finally increase the stock returns of the health care industry (37, 81).

The second mechanism is that air pollution changes investors' physiology and psychology (82, 83), which ultimately affect stock markets (10, 35, 36). Physiologically, air pollution is correlated with the level of cortisol, which has a negative influence on risk-taking behavior (73). Psychologically, air pollutants lead to bad moods and cognitive impairment (17, 19). First, people usually become pessimistic, tense and anxious in a polluted environment and tend to have negative expectations for listed companies' performance (17). Furthermore, they may mistakenly attribute their bad emotions to economics instead of air pollution, which also reduces their expectation for economic development (16, 18). Second, exposure to air pollution may damage mental attention, decrease sleep, increase logical confusion, and finally influence cognitive functioning (84–86).

## Quantile-on-Quantile Regression

We follow the work of Sim and Zhou (87) and show the QQ method as follows:


(1) $$\begin{array}{l} SR_{t}=\beta^{\theta }\left(AQ_{t}\right)+{\mu_{t}}^{\theta } \end{array}$$

where *AQ*_*t*_ and *SR*_*t*_ indicate the air quality and stock return at time *t*, respectively. β, θ and μ_*t*_ denote the parameter, θ*th* quantile and error term, respectively. We further linearize *β*^θ^(·) by taking a first-order Taylor expansion around *AQ*^τ^ (*AQ*^τ^ denotes the τ quantile of the OP). The new equation is shown as follows:


(2) $$\begin{array}{l} \beta^{\theta }\left(AQ_{t}\right)\approx \beta^{\theta }\left(AQ^{\tau }\right)+\beta^{\theta^{\prime }}\left(AQ^{\tau }\right)\left(AQ_{t}-AQ^{\tau }\right) \end{array}$$

where *β*^θ^′ is the partial derivative of $\beta^{\theta }\left(AQ_{t}\right)$ for *AQ*. However, the method presents a similar expansion for the slope parameter as in a linear regression model. Moreover, Equation (2) defines θ and τ as double indexed, and the parameters are indicated as $\beta^{\theta }\left(AQ_{t}\right)$ and $\beta^{\theta^{\prime }}\left(AQ_{t}\right)$, respectively. Hence, $\beta^{\theta }\left(AQ_{t}\right)$ and $\beta^{\theta^{\prime }}\left(AQ_{t}\right)$ are functions of θ and τ. Additionally, $\beta^{\theta }\left(AQ_{t}\right)$ and $\beta^{\theta^{\prime }}\left(AQ_{t}\right)$ are verified as β_0_(θ, τ) and β_1_(θ, τ), respectively. Thus, $\beta^{\theta }\left(AQ_{t}\right)$ is further described as follows:


(3) $$\begin{array}{l} \beta^{\theta }\left(AQ_{t}\right)\approx \beta_{0}\left(\theta ,\tau \right)+\beta_{1}\left(\theta ,\tau \right)\left(AQ_{t}-AQ^{\tau }\right) \end{array}$$

Then, we utilize Equation (3) to replace $\beta^{\theta }\left(AQ_{t}\right)$ in Equation (1) and obtain the following:


(4) $$\begin{array}{l} SR_{t}=\frac{\beta_{0}\left(\theta ,\tau \right)+\beta_{1}\left(\theta ,\tau \right)\left(AQ_{t}-AQ^{\tau }\right)}{*}+{\mu_{t}}^{\theta } \end{array}$$

The link between AQ and SR in each respective quantile is captured since the coefficients of β_0_ and β_1_ depend on θ and τ. In order to estimate Equation (4), *AQ*_*t*_ and *AQ*^τ^ are required instead with their counterparts ${\hat{AQ}}_{t}$ and ${\hat{AQ}}^{\tau }$, respectively. Therefore, the parameters *b*_0_ and *b*_1_ are estimated as the minimization problem in Equation (5):


(5) $$\begin{array}{l} min_{b_{0},b_{1}}\overset{N}{\underset{i=1}{\sum }}\rho \theta \left[SR_{t}-b_{0}-b_{1}\left({\hat{AQ}}_{t}-{\hat{AQ}}^{\tau }\right)\right]\\ \times K\left(\frac{F_{n}\left({\hat{AQ}}_{t}\right)-\tau }{h}\right) \end{array}$$

where ρθ(μ) is the absolute value function of the slope. *K*(·) is a Gaussian kernel. *h* indicates the bandwidth parameter of the kernel method and is equal to 0.05 in our paper. We also notice that the weights are inversely related to the distribution between ${\hat{AQ}}_{t}$ and ${\hat{AQ}}^{\tau }$, and the function is described as follows:


(6) $$\begin{array}{l} F_{n}\left(AQ_{t-1}\right)=\frac{1}{n}\overset{n}{\underset{k=1}{\sum }}I\left(AQ_{k}<AQ_{t-1}\right) \end{array}$$

Similarly, the same empirical steps are repeated to investigate the cases of stock returns in each industry, and the model permits us to test and verify the mentioned hypothesis.

This method can fully capture the potential asymmetric response of dependent variable across distributions of both explanatory and dependent variables. In addition, it can consistently estimate the smooth changing parameters and uncover underlying structural breaks in the real data. Last, since we consider the impacts of temporal-lag in the independent variable on the contemporaneous dependent variable, the QQ method ameliorates the endogeneity problem regarding the simultaneity which is contributed into the nature of its model specification. Depending on the mentioned strengthens, the QQ method has been widely employed in following fields, including agriculture commodity futures (88), stock market (46), oil market (89), industrial growth (90), and CO_2_ emission (91).

## Data and Empirical Results

### Data Source and Description

**Figure 2:**
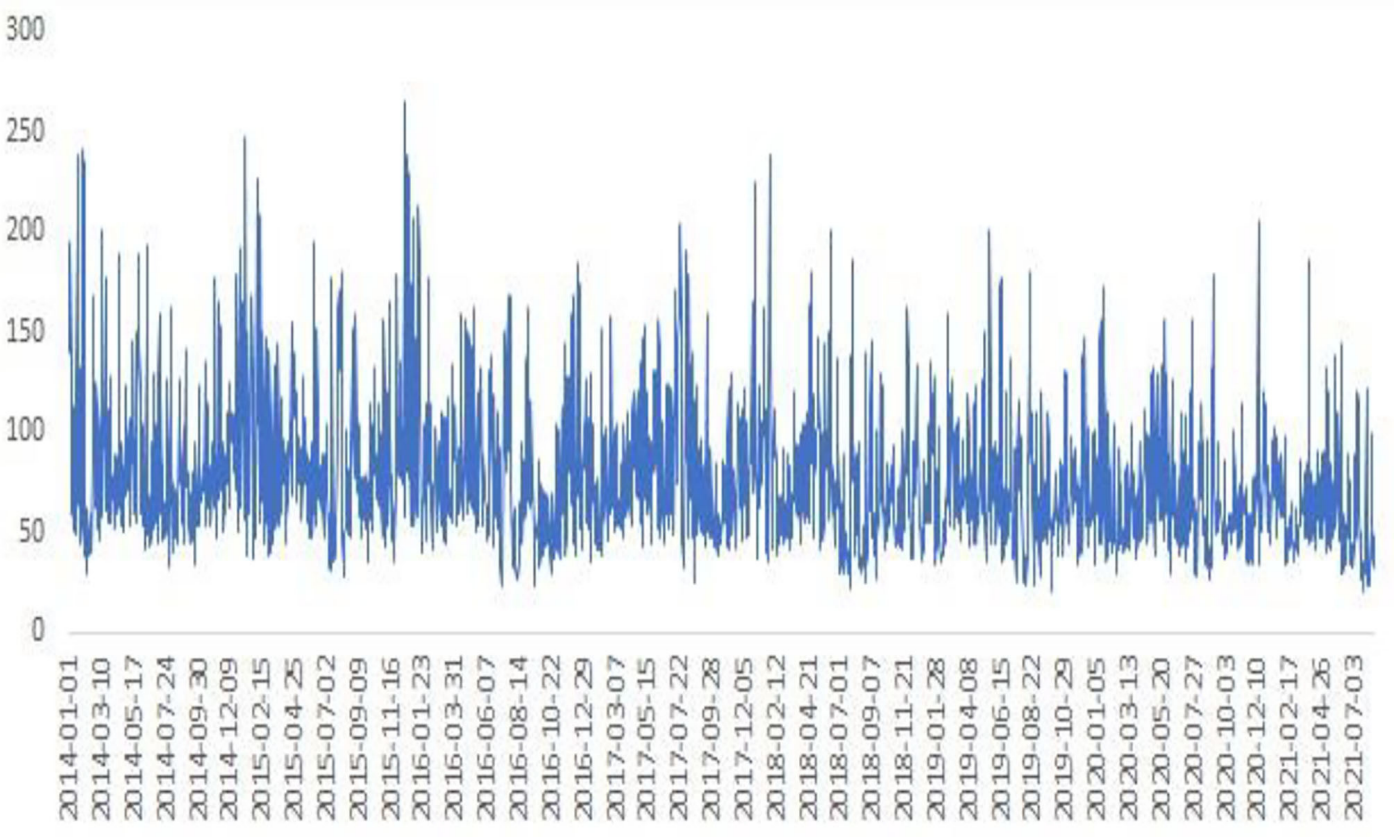
The trend of AQ index.

Our sample contains daily trading data that range from January 1, 2014 to July 31, 2021. Two types of variables are employed in this paper. The independent variable is Shanghai's air quality index (AQ). Shanghai is located in the Yangtze River Delta and has a subtropical monsoon climate. Shanghai is one of the most urbanized and modernized cities and is the economic center in China. The AQ index data come from the Ministry of Ecology and Environment of the People's Republic of China (MEEPRC), which has published daily data since January 2014. The AQ index can be divided into multiple levels, which is shown in Table 1. For example, when the values of AQ range from 0 to 100, the air quality is defined as fine and moderate, which does not cause the issue of air pollution. However, when the values are bigger than 100, the air quality is regarded as unhealthy and produce a significant influence on people's physiology and psychology. As shown in Figure 2, AQ presents huge volatility, and higher values usually emerge in spring and winter of the year. We provide potential reasons to explain this phenomenon. First, spring and winter are heating periods in northern China, and the major fuel is coal, which is one of the most polluting forms of energy. Second, at the end of the year, local governments tend to relax the requirement of environmental protection to reach their economic growth targets. The dependent variable is the stock return (SR). To comprehensively investigate the relationship between and SR, we choose four different health industries, which include health services (HS), health equipment (HE), health businesses (HB), and health technology (HT), as samples. The mentioned industries are tightly correlated with AQ, and their results could provide more valuable policy references. According to the traditional approach, this paper takes the logarithmic difference of the stock index to obtain SR, which can be described as SR_t_ = LnP_t_ -LnP_t−1_. In the formula, P_t_ and P_t−1_ indicate the stock indices at time t and t-1, respectively. Table 2 presents the descriptive statistics for all variables. The skewness values of all variables except AQ are <0, which indicates that the tail of the left side of the distribution is fatter than the tail on the right side. The kurtosis values of all variables are larger than 3, which demonstrates that their tails are fatter and obey a leptokurtic distribution. The Jarque-Bera test has the null hypothesis of a normal distribution and demonstrates that all variables reject the null hypothesis and follow a non-normal distribution.

**Table 1:** The AQ index classification levels.

**Air quality index (values)**	**Levels of health**	**Health concern & meanings**
0–50	Good	There is little risk and the air is not polluted. People are satisfied with this level of air quality.
51–100	Moderate	Most people find the air acceptable, yet a few people may worry that some pollutants in the air will cause health problems.
101–150	Unhealthy for sensitive groups	General people will not be seriously affected by this level of air quality, while people with lung disease are exposed with greater risks.
151–200	Unhealthy	Air quality of this level poses threats to physical health of everyone, and the sensitive groups face greater risks.
201–300	Very unhealthy	The health condition of every people is seriously affected by the mixture of pollutants in the air.
301–500	Hazardous	The air quality is intolerant for the entire population, which causes critical health warnings.

**Table 2:** Descriptive statistics of the variables.

	**Maximum**	**Minimum**	**Std. Dev**.	**Skewness**	**Kurtosis**	**Jarque-Bera**
AQ	266	24	35.032	1.393	5.506	1019.027^***^
HS	0.077	−0.089	0.021	−0.329	4.685	251.508^***^
HE	0.074	−0.091	0.021	−0.465	5.158	424.476^***^
HB	0.069	−0.093	0.017	−0.568	6.505	1043.922^***^
HT	0.095	−0.103	0.018	−0.747	9.049	2986.122^***^

*** *denotes significance at 1% level*.

### Empirical Results

**Figure 3:**
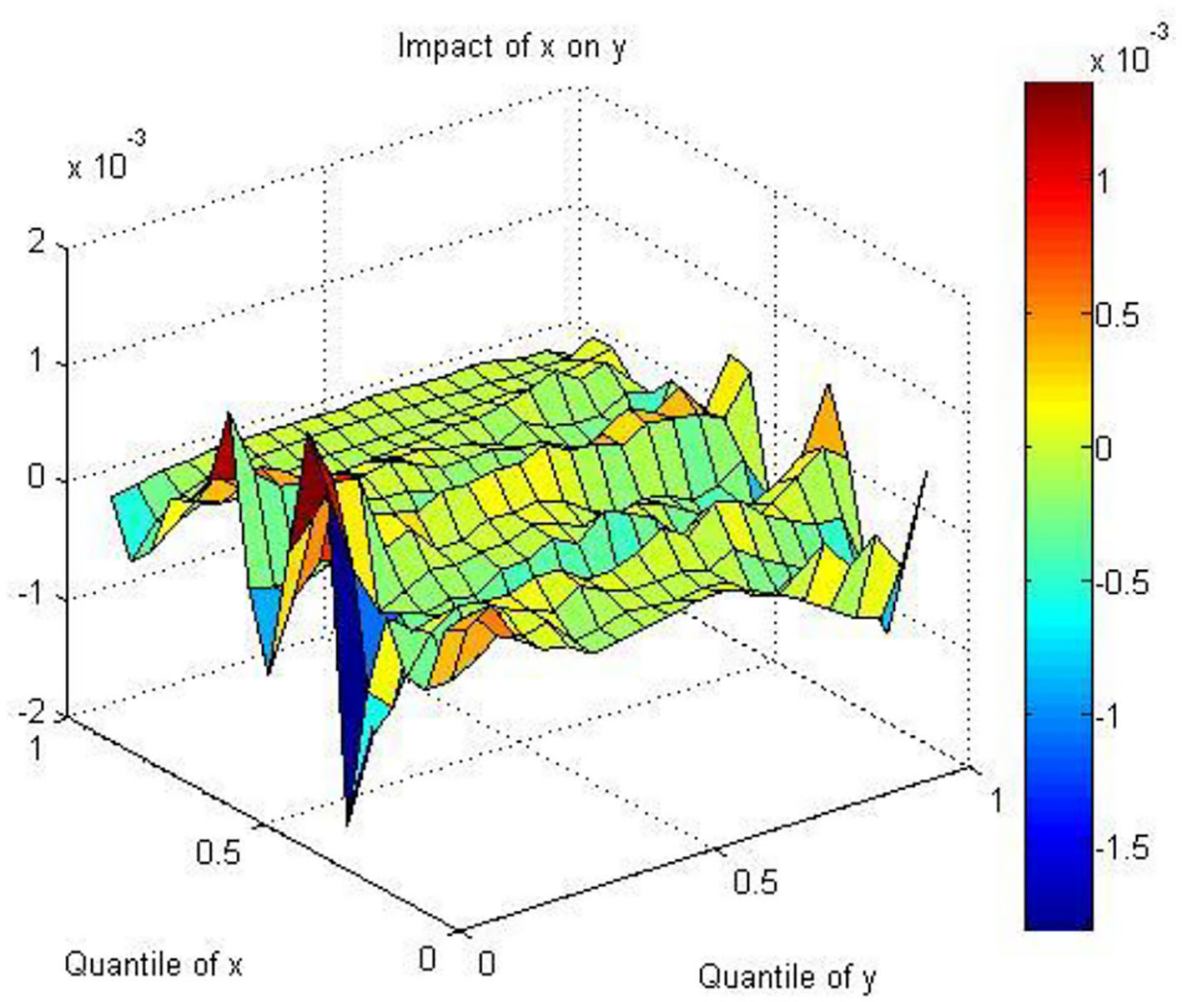
The coefficients between AQ and HS.

Figure 3 presents the results regarding the effect of air quality on the stock returns of HS using the QQ regression approach. As shown in the figure, the scale of the colored bar indicates the coefficients between AQ and HS, inferring the influence of different quantiles of AQ on health services. Dark blue and dark red indicate the lowest and highest values of the coefficients, respectively. We find that the coefficients are positive in the [0, 0.2] and [0.4, 0.8] quantiles and negative in the [0.2, 0.4] and [0.8, 1] quantiles for AQ. This demonstrates that good AQ decreases SR and bad AQ increases SR, which can be explained in the following ways. First, the phenomenon of an aging population in China is intensifying. The number of people over 60 years old has increased rapidly from 178 million in 2010 to 260 million in 2020, increasing from 13.3% of the total population in 2010 to 18.7% of the total population in 2020. Luo et al. (92) clearly state that long-term exposure to air pollution would damage mental abilities and episodic memory among middle-aged and old adults in China. Woodward and Levine (93) also indicate that older adults, accompanied by decreased cardiovascular function, experience higher mortality risks due to exposure to air pollution. Therefore, the increasing number of old adults in China brings great needs for health services. Second, China has published a series of policies to support the development of the health service industry. The National Health and Family Planning Commission (NHFPC) issued a document to effectively integrate social health services into the overall consideration of regional health planning. In 2015, the State Council released a document to further relax access to social health services and control the scale of public hospitals. Since then, many policies have been issued to encourage the access conditions, administrative examination and approval, investment and financing channels, hierarchical diagnosis and treatment, and expansion of high-quality societal hospitals. Since 2019, policy has emphasized supporting social health services in various specialized health service fields and accelerating the creation of a number of competitive brand service institutions in ophthalmology, orthopedics and other fields.

**Figure 4:**
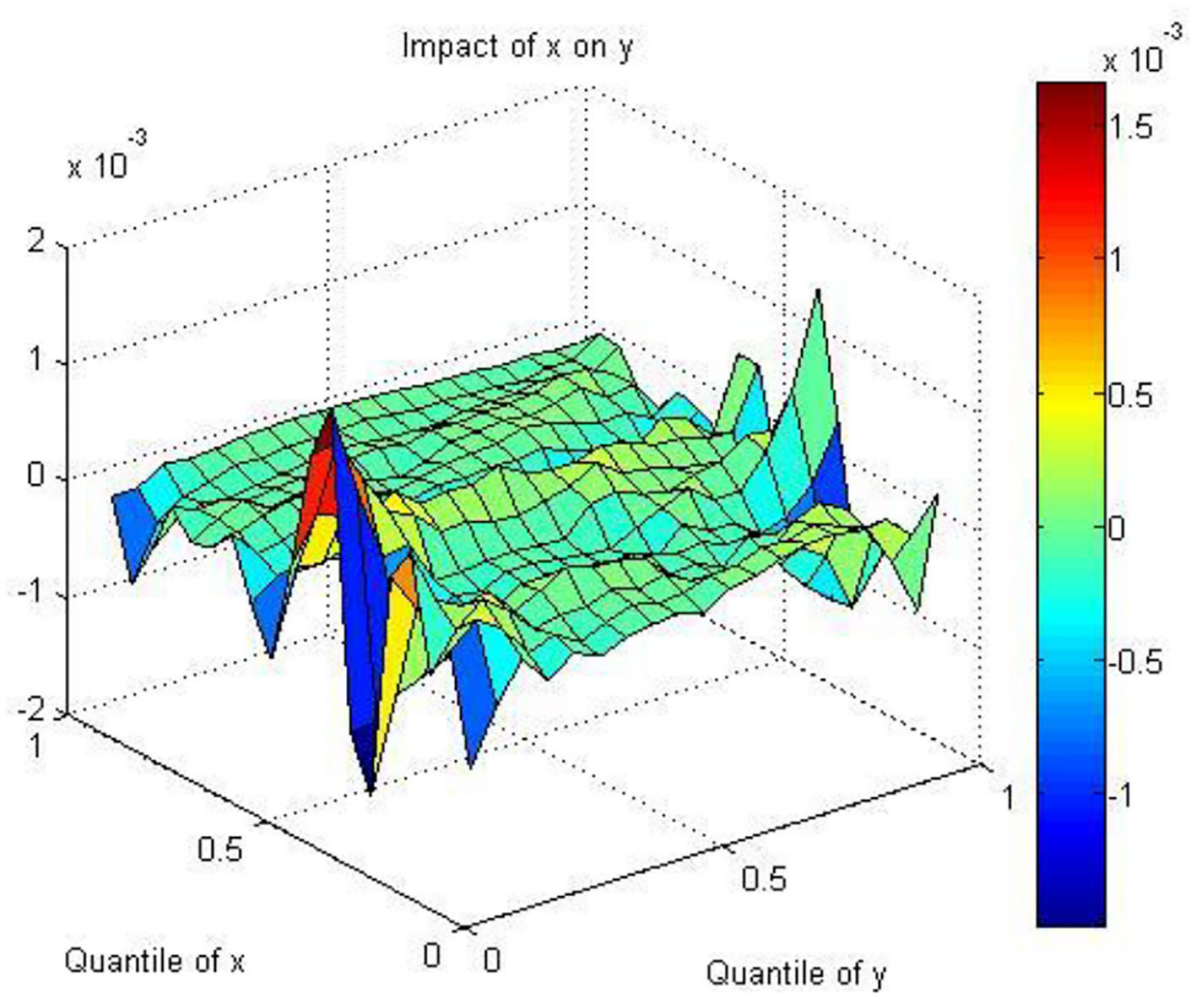
The coefficients between AQ and HE.

Figure 4 indicates that the coefficients between AQ and HE are negative in all quantiles of AQ, except in the [0.4, 0.5] quantile. Air pollution directly influences body organs and tissues, which results in respiratory disease, poor mental health, and subjective illness (64, 94). People are concerned about their health and reflect on the stock of the health industry through investors' behaviors. Therefore, the negative relationship between AQ and HE can be explained from physiological and psychological perspectives. Air pollution produces stressful stimuli; thus, people experience emotional, mental, and physical changes (95). Investors and other market participants are more pessimistic as air pollution increases and mistakenly attribute bad moods to depressed economic performance instead of air pollution (17, 96). This may lead to biased decisions and reduce stock purchases, which ultimately influence stock returns. In addition, government policy also plays a key role in explaining the negative relationship. Considering the health risks brought by air pollution, the government has proposed the concept of “Healthy China” to develop the health industry to reduce this type of risk. From 2010 to 2015, the total value of the health equipment of all of China's hospitals nearly doubled and reached 629 billion RMB (97). In 2020, the global health equipment market reached nearly 500 billion U.S. dollars, with a growth rate of nearly 4%. The scale of China's market is nearly 800 billion RMB, with a growth rate of nearly 20%, much higher than the global average. In the future, China is expected to cultivate a number of global health equipment leaders. First, the rapid growth of the domestic market will become important support for the rise of domestic leaders. Second, local equipment leaders are expected to continue to expand their shares in the international market. As shown by the NHFPC, China has proposed a strategy for developing modern health equipment with the purpose of constructing high-technology, large-scale, precise and valuable instruments. However, we should note that developing HE requires a long time, which indicates that bad air quality cannot influence the health equipment industry immediately.

**Figure 5:**
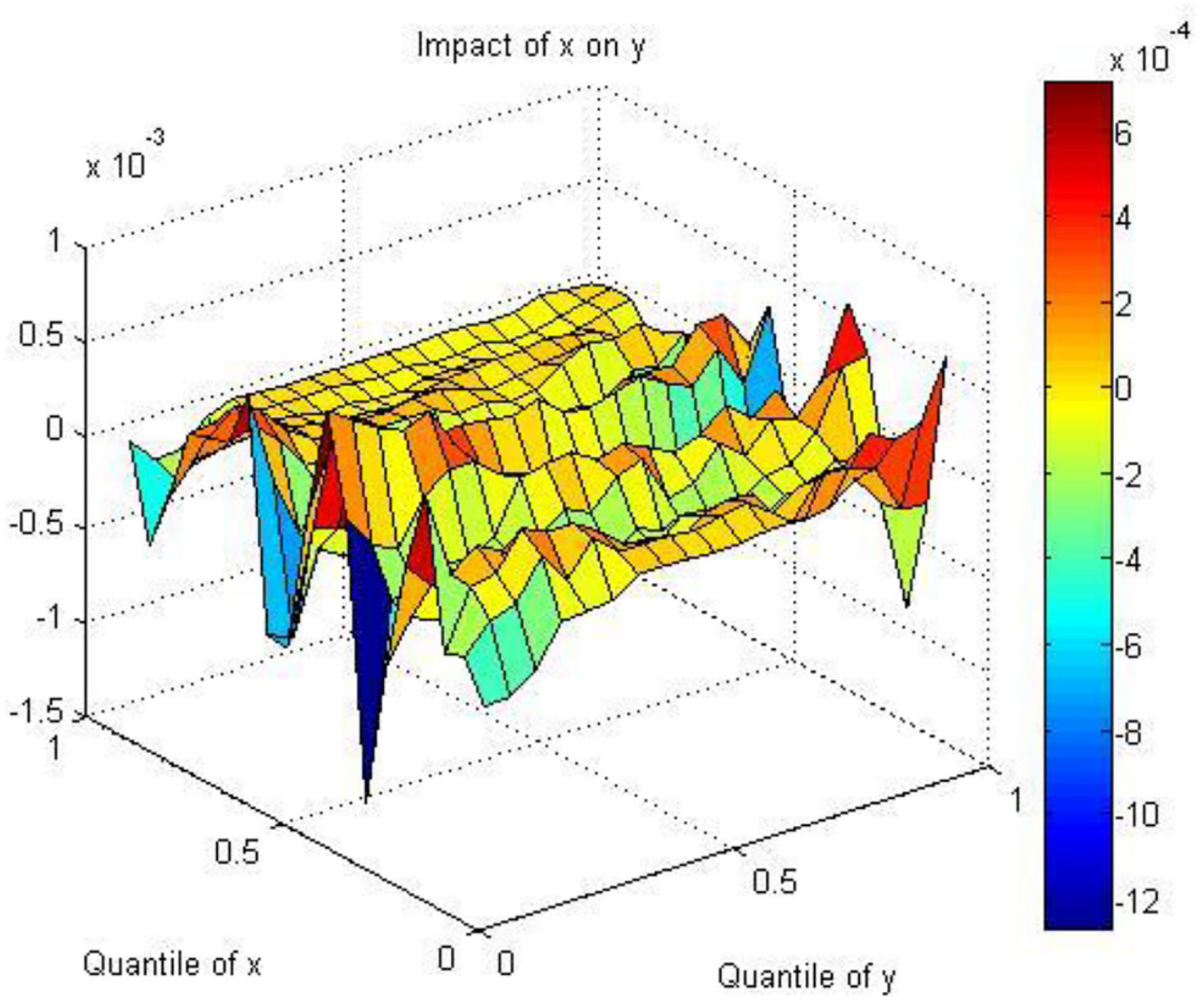
The coefficients between AQ and HB.

Figure 5 shows the coefficients between AQ and HB, and a positive relationship exists. Although a positive link exists in all quantiles of AQ, the values of the coefficients in the [0, 0.5] quantile are larger than those in the other quantiles. Air quality is able to bring changes in investors' physiology and psychology and further influences the stock market. Liu et al. (73) indicate that exposure to good air can trigger decreases in cortisol levels in humans, which makes people more likely to engage in risk-taking behaviors. Hence, good air quality would decrease investors' risk aversion and cause individual investors to purchase more stocks. Furthermore, health studies also find that exposure to good air quality increases spirits, concentration, relaxation and optimism, which are beneficial for performing cognitive functioning (19). Because trading decisions require strong cognitive function, cognitive functioning may lead investors to make the most appropriate trading decisions. However, in an environment with air pollution, the relationship between AQ and HB is still positive, which is mainly due to industrial policies rather than physiology and psychology. China has become the second largest health care market in the world. Depending on the prosperous economy, booming electronic commerce, and participating in the World Trade Organization, China's distribution and logistics systems in the health market have greatly improved (98). Since 2015, a number of policies related to drugs have been revised and newly published, which has had a significant influence on China's health care industry (99). In 2017, some key changes occurred, such as the new version of the National Reimbursement Drug List that determines which drugs are included in national health insurance. In addition, several new regulations were launched to change the clinical trial process, optimize the drug registration process, and manage drug prices. In addition, in recent years, China has also encouraged investments by leading health device companies, both foreign and domestic. The National Healthcare Security Administration (NHSA) and the National Health Commission (NHC) are promoting the reform of the “drug dual channel,” which aims to meet the reasonable needs of drug supply guarantees and clinical use through two channels, designated health institutions and designated retail pharmacies; and simultaneously incorporate the two channels into the payment mechanism of health insurance.

**Figure 6:**
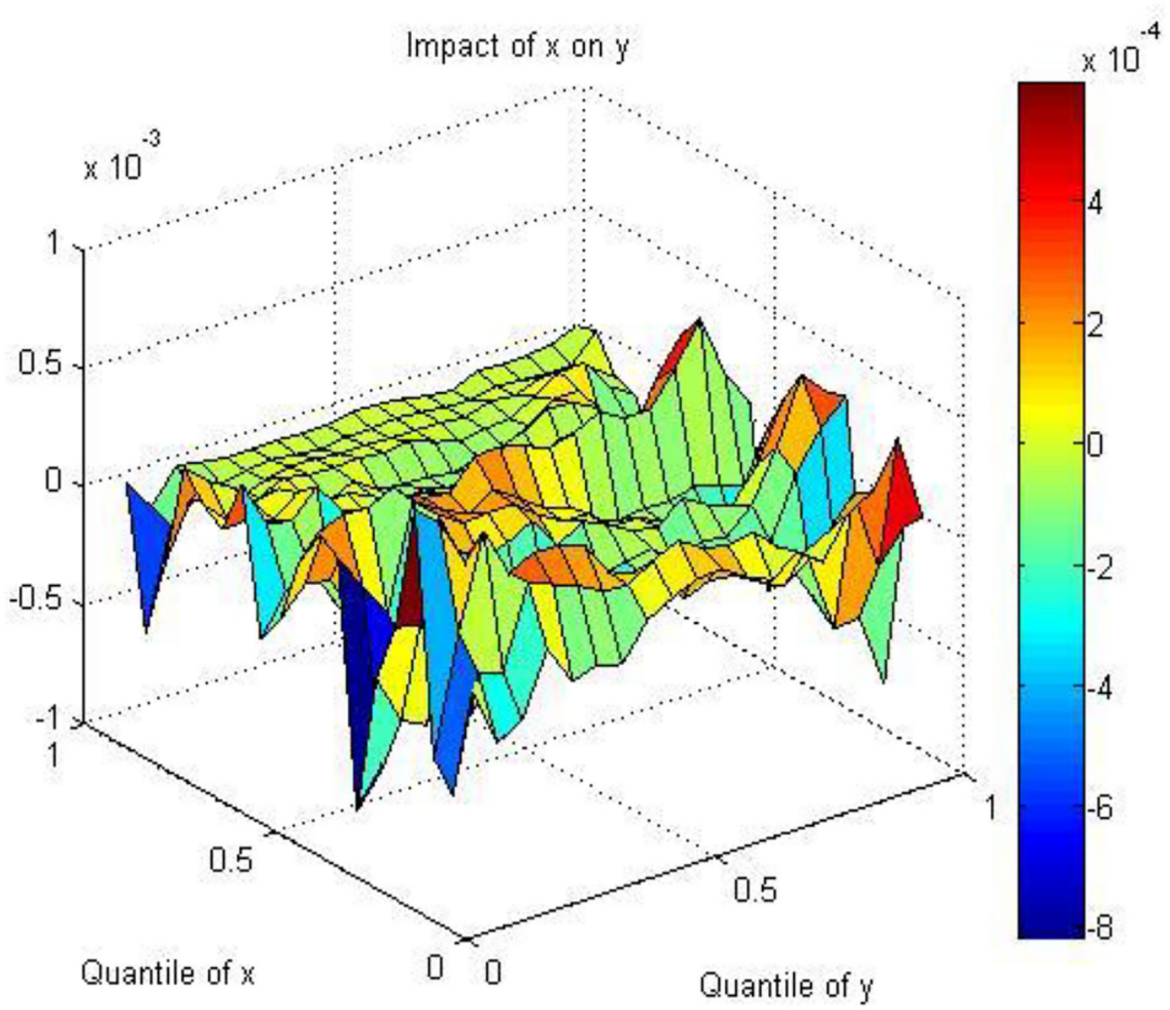
The coefficients between AQ and HT.

Figure 6 shows that the coefficients between AQ and HT are positive in the [0, 0.5] quantile and decrease and even become negative in the [0.5, 1] quantile of AQ. As explained in the health business industry, both physiological and psychological factors play important roles in this positive relationship. Some prior health studies have demonstrated that air quality significantly affects people's cognitive functions (7, 12, 34). Contact with good air can increase the ability of red blood cells to transfer oxygen to human organs, which is beneficial for concentrating spirit and avoiding confusion. Under this situation, people are more optimistic and relaxed and possess strong cognition, which makes them more likely to purchase stocks and engage in more stock trading (100). However, exposure to air pollution would have negative influences on stock markets in the following ways. First, a number of studies offer evidence that exposure to air pollution would damage the human brain and impair cognitive function, which has a negative influence on activities and decisions. Because stock trading involves cognitively demanding decisions, contact with polluted air damages investors' cognitive abilities. Second, air pollution can lead to mood changes for individuals; and moods are tightly related to investment decisions, asset valuation, and asset pricing in stock markets. In addition, long-term exposure to air pollution would result in sleep loss, which further brings the problems of judgment errors, attention loss, and inefficient information processing capabilities. Government industrial policies need time to play better roles in the health technology industry. In 2016, China's health care market grew by 10.6% to reach 1.5 trillion RMB and is estimated to reach 2 trillion RMB in 2021. Thus, China published a document of the guiding opinions on accelerating the structural adjustment of the health care industry, which includes promoting cross regional and cross ownership mergers and acquisitions and improving the economies of scale and industrial concentrations of companies. However, the health technology industry has the characteristics of high investments, high risk, high returns and a long cycle. The global health technology industry is increasingly showing a highly concentrated oligopolistic and monopolistic competition pattern. Bayer, Sanofi-Aventis, Merck, GlaxoSmithKline, Johnson & Johnson and fourteen other international health giants occupy 70% of the global market. Based on serious foreign competition, China's industrial supporting policies for health technology companies need time to take effect and thus affect stock markets in a timely manner.

Summarily, the link between air quality and stock returns present heterogeneous characters in different industries. For the HS and HT, good AQ decreases SR and bad AQ increases SR, which can be explained by healthcare demand. In addition, the coefficients between AQ and HE are negative in all quantiles of AQ, except in quantile of [0.4, 0.5]. Oppositely, the coefficients between AQ and HB, and there exists positive relationship. Given the complex relationship between AQ and SR in China's health industry, the government and health enterprises need to employ multiple policy tools, such as increasing R&D expenditure and improving health system, to main stable development of health industry and reduce shocks from changes in air quality.

## Conclusion and Policy Implications

This paper discusses the asymmetric link between air quality and stock returns for the health service (HS), health equipment (HE), health business (HB), and health technology (HT) industries. The major empirical results reveal that SR responds differently to AQ, which depends on the industry. First, the coefficients in HS and HT would change from positive to negative, but they have different turning points with respect to AQ. For example, the negative links for HS and HT are in the [0.8, 1] and [0.5, 1] quantiles, respectively. Second, the coefficients between AQ and HB have a positive relationship. Although a positive link exists in all quantiles of AQ, the values of the coefficients in the [0, 0.5] quantiles are larger than those in the other quantiles. Third, the coefficients between AQ and HE are negative in all quantiles of AQ, except in the [0.4, 0.5] quantile, which is different from other industries. When compared with previous literature, our study provides valuable contributions. First, we provide a new channel from government intervention to explain the influence of AQ on SR. China's government has launched a series of industrial structure upgrading guidelines; thus, these guidelines should be considered in explaining the relationship between AQ and SR. Second, industrial heterogeneity is fully considered in this study. Industries respond differently to the same external shock due to their industrial characteristics. Last, the quantile-on-quantile method, which can examine the effects of specific quantiles between variables without considering structural break and temporal lag effects, combines non-parametric estimation and the traditional quantile regression.

According to the conclusions, some policy implications are provided. First, investors, regulators and other market participants should notice that their biased decisions may be caused by air pollution instead of depressed economic development. Therefore, air quality should be regarded as an important factor in making cognitively demanding decisions in stock markets. Second, air pollutants seriously affect people's physiology, such as their cortisol levels; cognition and psychology, such as tension and anxiety. Hence, authorities should establish environmental protection law systems to decrease pollutant emissions and construct a strict mechanism for the disclosure of environmental information to the public. Third, China should optimize the structure of its health industry, encourage mergers and acquisitions across regions, construct independent health and medical innovation systems, and strengthen awareness of obeying laws and regulations among health companies. Besides, health education is needed to improve the residents' health literacy, such as how to make proper utilization of social security policies to protect themselves. More attention should be paid to vulnerable groups, such as rural, female, and old residents, to promote their equal access to basic public health service.

## Data Availability Statement

The original contributions presented in the study are included in the article/supplementary material, further inquiries can be directed to the corresponding author/s.

## Author Contributions

LL: conceptualization. K-HW: methodology and write. YX: visualization and investigation. All authors contributed to the article and approved the submitted version.

## Funding

This research has been supported by the Social Science Foundation of Shandong, China (Grant Number: 21DJJJ09).

## Conflict of Interest

The authors declare that the research was conducted in the absence of any commercial or financial relationships that could be construed as a potential conflict of interest.

## Publisher's Note

All claims expressed in this article are solely those of the authors and do not necessarily represent those of their affiliated organizations, or those of the publisher, the editors and the reviewers. Any product that may be evaluated in this article, or claim that may be made by its manufacturer, is not guaranteed or endorsed by the publisher.
